# The Value of Preoperative C-Reactive Protein to Albumin Ratio as a Prognostic Biomarker in Colon Cancer Patients

**DOI:** 10.3390/medicina60071054

**Published:** 2024-06-27

**Authors:** Giorgiana Fagarasan, Radu Seicean, Vasile Bintintan, Vlad Fagarasan, Alexandra Caziuc, David Andras, Lucian Chira, George Dindelegan

**Affiliations:** First Surgical Clinic, “Iuliu Hatieganu” University of Medicine and Pharmacy, 400012 Cluj Napoca, Romania; giorgianaamarinei@yahoo.com (G.F.); vbintintan@gmail.com (V.B.); vlad.fagarasan@yahoo.com (V.F.); alex.8610@gmail.com (A.C.); dr.andrasdavid@gmail.com (D.A.); chiralucianionut@gmail.com (L.C.); george.dindelegan@gmail.com (G.D.)

**Keywords:** colon cancer, C-reactive protein to albumin ratio, prognosis

## Abstract

Inflammatory acute phase proteins have been reported to play a crucial role in cancer progression. Various hematologic and inflammatory markers and scores, such as the lymphocyte-to-monocyte ratio, neutrophil-to-lymphocyte ratio, platelet-to-lymphocyte ratio, systemic inflammation score (SIS), prognostic nutritional index (PNI), Glasgow prognostic score, and, more recently, the Naples prognostic score, have been reported as significant prognostic markers. The aim of this prospective study was to evaluate the prognostic significance of the C reactive protein-to-albumin ratio (CAR) in patients with colon cancer. *Materials and Methods*: We conducted a prospective observational study on a series of patients who underwent curative surgery for colon cancer. The C reactive protein-to-albumin ratio was determined preoperatively, and we evaluated the correlations between the CAR and various clinical and pathological parameters, as well as the correlation with Overall and Relapse-free survival. Furthermore, we compared the accuracy of the CAR with that of the Naples score. *Results*: One hundred and ten patients were included in the study. We set 0.4927 as the cut-off value for the CAR according to a receiver operating characteristic curve analysis. Based on the cut-off value, patients were divided into a low CAR group and a high CAR group. The preoperative CAR exhibited statistically significant correlation with tumor volume, T and N stage, number of positive lymph nodes, and grade of tumor differentiation. We also demonstrated a positive correlation between high CAR values and a higher Naples score (*p* = 0.0005), even when a subgroup analysis was performed for each group individually. *Conclusions*: The preoperative CAR is a useful prognostic marker in patients with colon cancer. These results may help to design strategies to personalize targeted management approaches among colon cancer patients.

## 1. Introduction

Colon cancer represents the third-most frequently diagnosed malignant tumor globally and one of the main causes of cancer death worldwide, and it is associated with a high mortality rate despite an improvement in multidisciplinary approaches like radical and curative surgery and improvement in chemotherapy regimens. In Romania, the incidence of colon cancer is estimated to be 12.7%, representing the second-most frequent cause of death related to cancer [1]. The worldwide prognosis for colon cancer remains reserved, with 5-year survival rates of 65%. Despite recent advances in surgical procedures and adjuvant chemotherapy, a large number of patients relapse after curative surgery [2]. Although the TNM classification is useful for selecting the appropriate strategy for patients with colorectal cancer, the TNM staging system on its own cannot predict an accurate prognosis. That is why it is important to define novel biomarkers for selecting subgroups of patients who are most likely to have a poor prognosis.

Until recently, numerous biomarkers with a prognostic role in colon cancer and other various types of cancer have been described and investigated in the literature, such as the neutrophil-to-lymphocyte ratio (NLR), lymphocyte-to-monocyte ratio (LMR), platelet-to-lymphocyte ratio (PLR), Glasgow prognostic score (GPS), modified GPS (mGPS), and Naples score. Despite this fact, a significant number of the patients included in low-risk groups based on previously studied biomarkers present a paradoxical and fulminant evolution of the disease. This is why it is necessary to identify additional biomarkers, which may predict the prognosis of the disease more accurately and permit the selection of personalized and targeted oncologic or surgical treatment strategies. Despite the improvements in the multidisciplinary approach to colon cancer, the rate of mortality in this type of neoplasia is still significantly high, especially in metastatic or recurrent disease. The identification of strong biomarkers, which could predict prognosis or even recurrence, is of major clinical importance, with the goal of selecting patients who may benefit from intensive treatment regimens [3].

Tumor proliferation is conducive to tumor cell dissemination, leading to host reactions through the activation of inflammatory acute phase proteins [4]. Proinflammatory factors have a marked influence on tumorigenesis and tumor progression, as well as in the disease response to neoadjuvant and adjuvant treatment [5,6]. Despite the fact that the mechanisms underlying these processes are not fully understood, studies demonstrate a strong correlation between inflammation and tumorigenesis. The inflammatory response can promote tumorigenesis by affecting the tumor microenvironment and by releasing certain mediators like acute phase proteins, chemokines, and cytokines in the bloodstream, which further stimulate cellular proliferation and angiogenesis, exacerbating the aggressiveness of the tumor [7,8,9].

Recently, the C reactive protein-to-albumin ratio (CAR), which has been used for the purpose of predicting mortality in patients with sepsis, has been reported to correlate with survival in patients with hepatocellular carcinoma, esophageal squamous cell carcinoma, small cell lung cancer, and gastric cancer [10]. However, to the best of our knowledge, studies regarding the prognostic significance of the CAR in patients with colon cancer are scarce and have previously been conducted solely on Asian populations. The primary aim of our study was to evaluate the prognostic significance of the preoperative CAR by investigating if any correlations exist with the clinicopathological characteristics of the excised tumors and whether this translates into an influence on survival. The secondary aim of our study was to compare the accuracy of the CAR with other proposed biomarkers, such as the more extensively studied Naples score.

## 2. Materials and Methods

We conducted a longitudinal observational study on a series of consecutive patients diagnosed and treated for colon cancer at the First Surgical Department Cluj Napoca, County Emergency Clinical Hospital, between January 2021 and December 2023. We included, in this analysis, all cases of colon cancer who underwent curative surgery in the specified time period. Patients who received preoperative therapy, patients who underwent emergency surgery for bowel perforation and/or obstruction, and patients with metastatic disease and synchronous colic malignant disease were excluded from this study. Pretreatment staging methods consisted of colonoscopy with biopsy for confirmation of the tumor and computed tomography for the evaluation of the regional extent of the disease, as well as the exclusion of metastasis. All the resected specimens were pathologically classified according to the eighth edition of the UICC TNM classification system for malignant tumors. Tumor dimensions at the histopathological examination were recorded as height, width, and length and were expressed in centimeters. The total tumor volume was calculated by multiplying these parameters and was expressed in cm^3^. All patients were followed up regularly with physical and blood examinations, including measurements of the levels of tumor markers such as carbohydrate antigen 19-9 (CA19-9) and carcinoembryonic antigen (CEA), as well as mandatory screening using colonoscopy and computed tomography according to the ESMO guidelines.

Preoperative blood samples were obtained 1 day before surgery. Serum albumin is routinely determined for cancer patients, so this determination did not add to the costs of the hospital. The serum albumin and C reactive protein were measured using chemiluminescent immunoassay, according to the manufacturer’s protocol. The C reactive protein-to-albumin ratio was calculated from the preoperative blood samples by dividing the serum C reactive protein levels (expressed as mg/dL) by the serum albumin level (expressed as g/dL). In order to compare the CAR with additional biomarkers, the following parameters were also determined from blood samples: lymphocyte absolute value, monocyte absolute value, neutrophil absolute value, and total cholesterol levels (mg/dL). We defined the Naples score as described by a previous report [11] using the combination of serum albumin levels, total cholesterol, the lymphocyte-to-monocyte ratio, and the neutrophil-to-lymphocyte ratio. Patients with serum albumin <4.0 g/dL were allocated a score of 1, and >4 g/dL a score of 0. The threshold value for cholesterol level was 180 mg/dL. Values below this value were allocated one point, while values exceeding it were provided zero points. The neutrophil-to-lymphocyte ratio threshold was 2.96 and the lymphocyte-to-monocyte ratio was 4.4. Values above the specified thresholds were provided one point, while values less than the specified amount were provided zero points. Patients with scores of 0 were allocated to Group 0, patients with one-to-two points were allocated to Group 1, and patients with three-to-four points were allocated to Group 2. Overall survival was defined as the time elapsed between diagnosis of the tumor as evidenced by histopathological analysis of the biopsy specimen harvested during the initial colonoscopy and the time of patient death. Recurrence was identified by computed tomography scan or PET scan during adjuvant treatment or follow-up. Relapse-free survival was defined as the time elapsed between diagnosis through colonoscopy and diagnosis of the tumor recurrence during postoperative follow-up by the oncologist.

Statistical analysis was performed using GraphPad Prism Version 9.3.0, GraphPad Software, San Diego, CA, USA. Descriptive statistics were calculated as means with 95% confidence interval for continuous variables. Categorical variables were expressed through contingency tables. An analysis of the receiver operating characteristic (ROC) curve to determine the appropriate cut-off value for the C reactive protein/albumin ratio was used. All patients were classified into two groups according to this preoperative ratio (low CAR and high CAR). The significance of the correlation between the preoperative CAR and the tumor clinicopathological characteristics was analyzed using the χ^2^ test and *t*-test, based on postoperative pathology reports. The duration of the survival was calculated according to the Kaplan–Meier method. Differences in the survival curves were assessed with the log-rank test. Statistical significance was set at a value of *p* < 0.05.

This research conformed to the provisions of the Declaration of Helsinki in 1975. All patients were informed of the investigational nature of this study and provided their written informed consent. This prospective study was approved by the Ethics Committee of University of Medicine and Pharmacy Cluj Napoca “Iuliu Hatieganu” (DEP186/09.06.2023).

## 3. Results

Data were collected from the hospital database on 194 patients who underwent colon resection in the specified time period. One hundred and ten cases met the inclusion criteria, had complete data sets and were included for analysis. The demographic data of the patients and the tumor characteristics as evidenced by the histopathological examination are listed in Table 1. The median age of patients was 67.25 years old, ranging from 42 to 90 years old.

**Figure 1:**
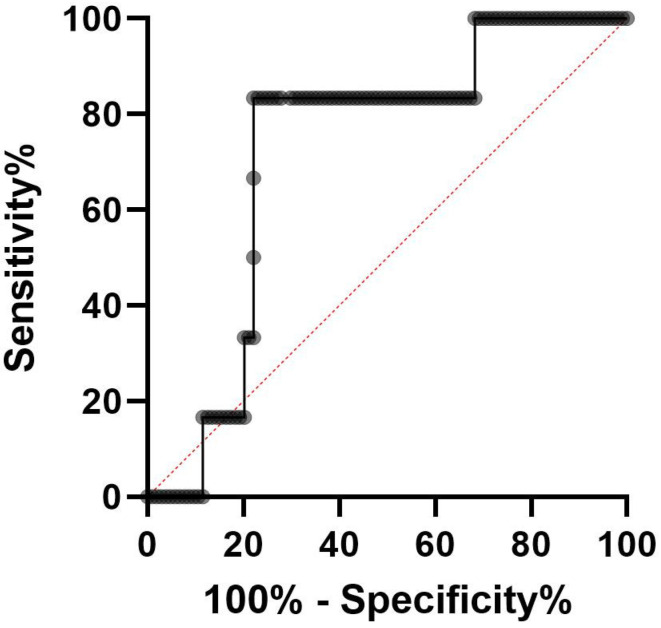
ROC curve analysis of C reactive protein to albumin ratio.

The median serum CRP value obtained was 1.81 mg/dL, ranging from 0.05 to 12.58. The median serum albumin value was 3.92 g/dL, ranging from 2.94 to 4.72. The median preoperative CAR was 0.5013, with a range from 0.012 to 3.639 (95% CI 0.3597–0.6428). ROC curve analysis demonstrated an appropriate cut-off value for the preoperative CAR to be 0.4927 (sensitivity of 83.33%, specificity of 77.88%) (Figure 1, Table 2). According to the cut-off value obtained, 82 patients were classified into the low CAR group and 28 patients were classified into the high CAR group.

The correlations between the preoperative CAR and clinicopathological parameters are shown in Table 3.

The preoperative CAR exhibited statistically significant relationships with tumor volume, with T stage, N stage, number of positive lymph node, and with the grade of differentiation. A high CAR was associated with more advanced tumor stages, T4 (*p* = 0.0392) and N2b (*p* = 0.0120). A high CAR was also associated with more frequent tumor deposits in the subserosa, mesentery, and nonperitonealized pericolic tissues (N1c- *p* = 0.0304). Lastly, a high CAR was associated with a low tumor differentiation grade. A higher C reactive protein albumin ratio was also associated with a higher number of positive lymph nodes (*p* = 0.0221).

The Naples score was calculated, and seven patients had a score of 0 and were included in Group 0, while 49 had a score of 1 or 2 and were classified in Group 1. Fifty-four patients had a score of 3 or more and were included in Group 2. Statistical analysis demonstrated a positive correlation between high CAR values and a higher Naples score (*p* = 0.0005), even when subgroup analysis was performed for each group individually. The results of the correlation analysis between Naples score and CAR are presented in Table 4.

**Figure 2:**
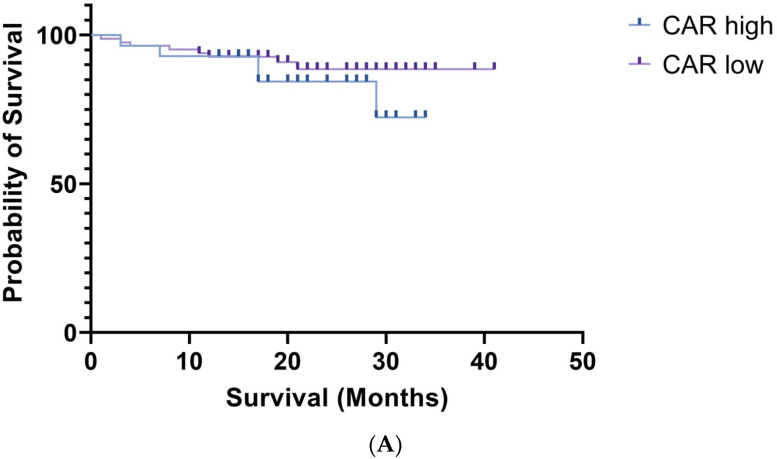

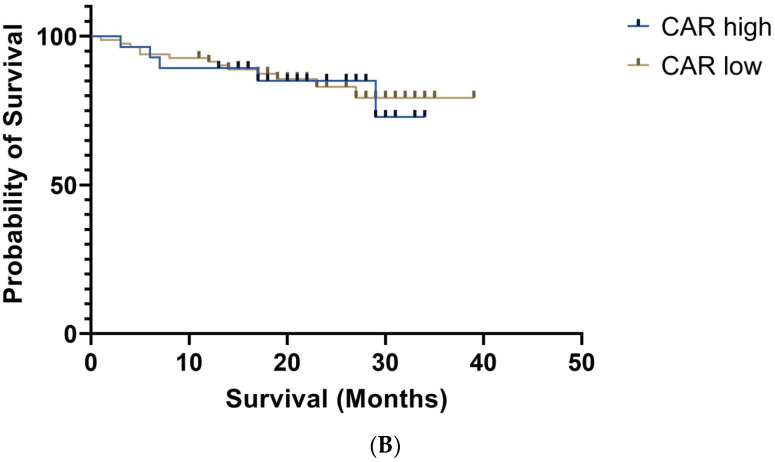
(**A**) Kaplan–Meier curves of overall survival for high CAR and low CAR groups; (**B**) Kaplan–Meier curves of relapse-free survival for high CAR and low CAR groups.

The results of the survival analysis are presented in Figure 2.

Patients in the high CAR group had a 1-year overall survival of 92.85% and a 2-year overall survival of 84.41%. In the low CAR group, 1-year overall survival was 92.66% and 2-year overall survival was 88.52%. There were no statistically significant results when comparing the overall survival of the two groups (*p* = 0.2945) using the Wilcoxon and log-rank tests. Relapse-free survival for the high CAR group was 89.28% at 1 year of follow-up and 85.03% at 2 years of follow-up. In the low CAR group, 1-year relapse-free survival was 91.44% and 2-year relapse-free survival was 82.96%. The observed differences in relapse-free survival for these groups were not statistically significant (*p* = 0.8612).

## 4. Discussion

An increasing number of studies suggest a close relationship between inflammation, tumorigenesis, and angiogenesis [12,13]. Inflammatory response can promote tumor progression by altering the tumor microenvironment, leading to the production of various chemical mediators such as acute phase proteins, chemokines, and cytokines [14,15,16]. In recent decades, some peripheral inflammatory markers like the neutrophil-to-lymphocyte ratio and platelet-to-lymphocyte ratio have been reported to correlate with the prognosis of patients with various types of malignancies and have been used to predict colorectal cancer outcomes [17].

C reactive protein is an acute-phase inflammatory protein that is synthesized by hepatocytes, its serum levels being regulated by proinflammatory cytokines such as interleukin IL-1, IL-6, and tumor necrotic factor TNF-α [18]. The serum albumin concentration is not only an objective parameter of nutritional status but is also associated with chronic inflammation. Under conditions of inflammation, the production of albumin is suppressed due to the activation of inflammatory cytokines, including IL-1, IL-6, and TNFα [19,20]. Thus, a combination of these two acute phase proteins, namely the C reactive protein-to-albumin ratio, may more accurately reflect the severity of inflammation, which is believed to be directly correlated with tumor progression.

The Naples score has been reported to be a useful prognostic tool in colorectal cancer patients who have undergone curative surgery. The Naples score is a simple scoring system that is easy to calculate and is based on the serum albumin, total cholesterol, neutrophil-to-lymphocyte ratio, and lymphocyte-to-monocyte ratio. In a recent study, Sugimoto et al. showed that a high Naples score is an independent prognostic factor for overall survival and disease-free survival for colon colorectal cancer patients undergoing curative resection. They concluded that the Naples score may be a helpful tool for optimization and personalization of adjuvant chemotherapy for colorectal cancer [11]. Our study demonstrated that there is a positive correlation between high CAR values and a higher Naples score, suggesting that CAR might also be an effective prognostic biomarker in colon cancer.

A recent meta-analysis by Qiang-ping Zhou et al. [21] has demonstrated that elevated pre-treatment C reactive protein-albumin ratio is associated with poor overall survival in patients with colorectal cancer. These conclusions were also supported by a similar study that concluded that a high C reactive protein–albumin ratio is associated with poor overall survival, disease-free survival, and progression-free survival in colorectal cancer, and that it can be useful as a prognostic marker for colorectal cancer in clinical practice. However, a major limitation of the meta-analysis was the retrospective design and the existence of comorbidities influencing the C reactive protein and the albumin values [22]. In our study, correlations were established between a high CAR and several clinicopathological tumoral parameters, such as the T and N stages, the number of positive lymph nodes, and the grade of differentiation. This indicates that the CAR does not simply reflect cancer progression but might be an independent prognostic factor reflecting the inflammatory status of the patient. Therefore, the combination of the TNM staging system and the CAR may contribute to predicting the prognosis and decisions regarding the selection of therapeutic strategies like adjuvant or even neoadjuvant chemotherapy for colon cancer more accurately, as there is no standardization for strict indications of neoadjuvant chemotherapy in colon cancer [23]. Despite the differences in overall and relapse-free survival observed in our study, which are consistent with previously published data, the small sample size did not allow us to draw statistically significant conclusions on the association between CAR and survival parameters.

The main strength of the present study is the fact that we included only colon cancer patients while excluding rectal cancer patients from our study, thus establishing a more homogenous group of patients and also factoring in the possibility of using this ratio for standardizing the indications for neoadjuvant chemotherapy in colon cancer. It is important to identify patients with a poor prognosis because neoadjuvant chemotherapy indications in the treatment of colon cancer are not sufficiently standardised. By identifying cases with poor prognosis, we may be able to apply more personalised treatment strategies in order to improve future outcomes for these patients. In addition, to the best of our knowledge, the C reactive protein-to-albumin ratio as a prognostic factor for colon cancer has previously been studied exclusively in Asian countries on a more heterogenous population, which included both colon and rectal cancer [24,25]. Therefore, our study comprises a novel population of patients, treated in a European tertiary care center, with a more focused approach towards patient selection. The primary limitation associated with this study is the small sample size, which does not allow us to draw firm conclusions in assessing the correlations between the investigated parameters. Also, potential confounding factors that may affect the CAR by lowering serum albumin levels, such as the presence of underlying comorbidities like liver cirrhosis or chronic renal failure, were not assessed. Despite these limitations, our findings suggest that a high C reactive protein-to-albumin ratio is associated with unfavorable clinicopathological tumor characteristics and that this ratio correlates with established scores, which have been previously assessed in the literature. However, additional multicentric studies based on a larger number of cases are required in order to further clarify these issues.

## 5. Conclusions

C reactive protein and albumin serum levels are very accessible and cost-effective biomarkers, which may have prognostic utility for colon cancer patients. A high CAR may be associated with unfavorable pathological tumor characteristics. Further multicentric studies on these readily available serum biomarkers are required in order to validate their clinical utility as prognostic indicators.

## Figures and Tables

**Table 1:** Patient characteristics.

Characteristic	Value (Percentage)
Gender, n	
Male	57 (51.81%)
Female	53 (48.18%)
Median tumor volume (cm^3^)	43.93 (0.48–1125)
Location of primary tumor	
Cecum	11 (10%)
Ascending colon	24 (21.81%)
Transverse colon	22 (20%)
Descending colon	16 (14.54%)
Sigmoid	37 (33.63%)
T stage, n	
Tis	2 (1.81%)
T1	5 (4.54%)
T2	18 (16.36%)
T3	61 (55.45%)
T4	24 (21.81%)
Tumor grade	
G1	32 (29.09%)
G2	73 (66.36%)
G3	5 (4.54%)
Lymphatic involvement, n	
Negative	75 (68.18%)
Positive	35 (31.82%)
Venous involvement, n	
Negative	91 (82.72%)
Positive	19 (17.27%)
Perineural involvement, n	
Negative	89 (80.9%)
Positive	21 (19.1%)
N stage, n	
N0	57 (51.81%)
N1a	11 (10%)
N1b	11(10%)
N1c	9 (8.18%)
N2a	11 (10%)
N2b	11 (10%)

**Table 2:** Sensitivity and specificity of CAR threshold value.

Threshold	Sensitivity%	95% CI	Specificity%	95% CI	Likelihood Ratio	*p*-Value	AUC
>0.4927	83.33	43.65% to 99.15%	77.88	69.00% to 84.79%	3768	0.00673	0.7228

**Table 3:** Multivariate analysis of CAR and histopathological parameters. * means that the result is statistically significant.

Parameter	CRP-to-Albumin Ratio		*p*-Value
	**Low**	**High**	
***Tumor location***			
Cecum	8	3	0.8840
Ascending colon	18	6	0.9539
Transverse colon	16	6	0.8267
Descending colon	9	7	0.0692
Sigmoid colon	31	6	0.1133
***Tumor volume (cm^3^)***	35.76 95%CI (7.38–64.13)	67.28 95%CI (38.15–96.4)	0.0008 *
***pT stage***			
T1	5	2	0.8449
T2	15	3	0.3493
T3	48	13	0.2657
T4	14	10	0.0392 *
***pN stage***			
N0	42	13	0.6616
N1a	9	2	0.5594
N1b	8	3	0.8840
N1c	5	4	0.0304 *
N2a	10	1	0.4149
N2b	6	5	0.0120 *
***Number of harvested lymph nodes***	18.45	22.04	0.0737
***Number of positive lymph nodes***	1.63	4.35	0.0221 *
***Lymphatic invasion***	23	11	0.1490
***Venous invasion***	10	9	0.6255
***Perineural invasion***	15	6	0.7185
***Tumor budding***	14	6	0.6059
***Differentiation***			
G1	27	4	0.0584
G2	54	19	0.8464
G3	1	4	0.0042 *

**Table 4:** Correlation between Naples score and CAR. * means that the result is statistically significant.

Naples Group	CRP to Albumin Ratio		*p*-Value
	Low	High	
**0**	6	1	0.4833
**1**	44	5	0.0010 *
**2**	32	22	0.0003 *

## Data Availability

Data is unavailable due to privacy or ethical restrictions.

## References

[1] Sung H., Ferlay J., Siegel R.L., Laversanne M., Soerjomataram I., Jemal A., Bray F. (2021). Global cancer statistics: GLOBOCAN estimates of incidence and mortality worldwide for 36 cancers in 185 countries. CA Cancer J. Clin..

[2] Haggar F.A., Boushey R.P. (2009). Colorectal cancer epidemiology: Incidence, mortality, survival, and risk factors. Clin. Colon Rectal Surg..

[3] Yamamoto T., Kawada K., Obama K. (2021). Inflammation-Related Biomarkers for the Prediction of Prognosis in Colorectal Cancer Patients. Int. J. Mol. Sci..

[4] Schmitt M., Greten F.R. (2021). The inflammatory pathogenesis of colorectal cancer. Nat. Rev. Immunol..

[5] Gunawardene A., Dennett E., Larsen P. (2019). Prognostic value of multiple cytokine analysis in colorectal cancer: A systematic review. J. Gastrointest. Oncol..

[6] Mantovani A., Allavena P., Sica A., Balkwill F. (2008). Cancer-related inflammation. Nature.

[7] Zhao H., Wu L., Yan G., Chen Y., Zhou M., Wu Y., Li Y. (2021). Inflammation and tumor progression: Signaling pathways and targeted intervention. Signal Transduct. Target. Ther..

[8] Greten F.R., Grivennikov S.I. (2019). Inflammation and Cancer: Triggers, Mechanisms, and Consequences. Immunity.

[9] Antonucci L., Karin M. (2024). The Past and Future of Inflammation as a Target to Cancer Prevention. Cancer Prev. Res..

[10] Alkurt E.G., Durak D., Turhan V.B., Sahiner I.T. (2022). Effect of C-Reactive Protein-to-Albumin Ratio on Prognosis in Gastric Cancer Patients. Cureus.

[11] Sugimoto A., Fukuoka T., Shibutani M., Kasashima H., Kitayama K., Ohira M., Maeda K. (2023). Prognostic significance of the Naples prognostic score in colorectal cancer patients undergoing curative resection: A propensity score matching analysis. BMC Gastroenterol..

[12] Hart P.C., Rajab I.M., Alebraheem M., Potempa L.A. (2020). C-Reactive Protein and Cancer-Diagnostic and Therapeutic Insights. Front. Immunol..

[13] Idos G.E., Kwok J., Bonthala N., Kysh L., Gruber S.B., Qu C. (2020). The Prognostic Implications of Tumor Infiltrating Lymphocytes in Colorectal Cancer: A Systematic Review and Meta-Analysis. Sci. Rep..

[14] Zhou X., Du Y., Huang Z., Xu J., Qiu T., Wang J., Wang T., Zhu W., Liu P. (2014). Prognostic value of PLR in various cancers: A meta-analysis. PLoS ONE.

[15] Li M.-X., Liu X.-M., Zhang X.-F., Zhang J.-F., Wang W.-L., Zhu Y., Dong J., Cheng J.-W., Liu Z.-W., Ma L. (2014). Prognostic role of neutrophil-to-lymphocyte ratio in colorectal cancer: A systematic review and meta-analysis. Int. J. Cancer.

[16] Chan J.C., Chan D.L., Diakos C.I., Engel A., Pavlakis N., Gill A., Clarke S.J. (2017). The Lymphocyte-to-Monocyte Ratio is a Superior Predictor of Overall Survival in Comparison to Established Biomarkers of Resectable Colorectal Cancer. Ann. Surg..

[17] Wu M.T., He S.Y., Chen S.L., Li L.F., He Z.Q., Zhu Y.Y., He X., Chen H. (2019). Clinical and prognostic implications of pretreatment albumin to C-reactive protein ratio in patients with hepatocellular carcinoma. BMC Cancer.

[18] Park J.W., Chang H.J., Yeo H.Y., Han N., Kim B.C., Kong S.-Y., Kim J., Oh J.H. (2020). The relationships between systemic cytokine profiles and inflammatory markers in colorectal cancer and the prognostic significance of these parameters. Br. J. Cancer.

[19] Han L., Guo Y., Ren D., Hui H., Li N., Xie X. (2023). A predictive role of C-reactive protein in colorectal cancer risk: An updated meta-analysis from 780,985 participants and 11,289 cancer cases. Int. J. Color. Dis..

[20] Christina N.M., Tjahyanto T., Lie J.G., Santoso T.A., Albertus H., Octavianus D., Putri D.A.U.I., Andrew J., Jatinugroho Y.D., Shiady C. (2023). Hypoalbuminemia and colorectal cancer patients: Any correlation?: A systematic review and meta-analysis. Medicine.

[21] Zhou Q.P., Li X.J. (2019). C-Reactive Protein to Albumin Ratio in Colorectal Cancer: A Meta-Analysis of Prognostic Value. Dose Response.

[22] Liao C.K., Yu Y.L., Lin Y.C., Hsu Y.J., Chern Y.J., Chiang J.M., You J.F. (2021). Prognostic value of the C-reactive protein to albumin ratio in colorectal cancer: An updated systematic review and meta-analysis. World J. Surg. Oncol..

[23] Morton D., Seymour M., Magill L., Handley K., Glasbey J., Glimelius B., Palmer A., Seligmann J., Laurberg S., Murakami K. (2023). Preoperative Chemotherapy for Operable Colon Cancer: Mature Results of an International Randomized Controlled Trial. J. Clin. Oncol..

[24] Shibutani M., Maeda K., Nagahara H., Iseki Y., Ikeya T., Hirakawa K. (2016). Prognostic Significance of the Preoperative Ratio of C-Reactive Protein to Albumin in Patients with Colorectal Cancer. Anticancer Res..

[25] Tamagawa H., Aoyama T., Numata M., Maezawa Y., Kazama K., Astumi Y., Hara K., Kano K., Yukawa N., Saeki H. (2021). Prognostic significance of the preoperative C-reactive protein-to-albumin ratio in patients with colorectal cancer. J. Cancer Res. Ther..

